# Case report: Response to Savolitinib/EGFR-TKI combination in NSCLC patients harboring concurrent primary MET amplification/overexpression and EGFR mutation

**DOI:** 10.3389/fonc.2024.1297156

**Published:** 2024-02-06

**Authors:** Xiaolin Ren, Kejie Li, Yang Zhang, Changlin Zou, Meng Su

**Affiliations:** Department of Radiation Oncology, The First Affiliated Hospital of Wenzhou Medical University, Wenzhou, Zhejiang, China

**Keywords:** primary MET amplification, MET overexpression, EGFR mutation, Savolitinib, EGFR-TKI, non-small cell lung cancer (NSCLC)

## Abstract

Lung cancer is the leading cause of cancer death, accounting for one-third of all cancer deaths worldwide. The MET (c-MET) gene, as one of the therapeutic target spots of NSCLC, has become increasingly more important. MET amplification/overexpression was divided into primary (intrinsic) and secondary (acquired). Studies indicated that the combination of Osimertinib and Savolitinib was safe and showed promising antitumor effect in NSCLC patients with secondary MET amplification after EGFR mutations. However, NSCLC patients with primary MET amplification/overexpression and EGFR mutations are rare in clinics, and the efficacy of dual-target therapy combined with EGFR-TKI and Savolitinib for them has not been studied yet. Here, we reported two NSCLC patients with primary MET amplification/overexpression and EGFR mutation, who benefited from T+S therapy (the dual-target therapy of EGFR-TKI plus Savolitinib) and achieved a progression-free survival (PFS) of approximately 5 months. The two cases indicated that T+S therapy has an acceptable safety profile and encouraging antitumor efficacy in NSCLC patients harboring concurrent primary MET amplification/overexpression and EGFR mutation. Meanwhile, the observation stresses the importance of genetic testing, and the MET gene needs to be detected at first diagnosis for the best choice of targeted therapies.

## Introduction

Lung cancer is the most common cancer mortality cause for both men and women, accounting for one-third of all cancer deaths worldwide. Small cell lung cancer (SCLC) and non-small cell lung cancer (NSCLC) are two major subtypes of lung cancer. Despite significant efforts made to fight lung cancer globally over the past few decades, the 5-year survival rate for NSCLC, which constitutes over 80% of lung cancer cases, is low at approximately 18% (1). The critical finding of EGFR-activating mutations led to the creation of epidermal growth factor receptor tyrosine kinase inhibitors (EGFR-TKIs), which is a key step in the targeted treatment of NSCLC. Additionally, the MET (c-MET) gene is another crucial therapeutic target for NSCLC and is strongly associated with survival, prognosis, and some treatment resistance of patients (2).

The gene mesenchymal epithelial transition (MET) is a proto-oncogene located on the long arm of chromosome 7 and contains 21 exons. It encodes a receptor tyrosine kinase protein known as hepatocyte growth factor receptor (HGFR), which, combined with the hepatocyte growth factor (HGF), activates downstream cell signaling pathways, and plays a role in cell proliferation, survival, and migration (3, 4). Dysregulation of the MET pathway in lung cancer happens through a variety of mechanisms, including MET kinase domain mutation, MET amplification, MET overexpression, and MET fusions. MET amplification/overexpression was divided into primary (intrinsic) and secondary (acquired). Studies have proved that secondary MET amplification serves as a mechanism of acquired resistance in patients with advanced NSCLC with EGFR mutation (5, 6). Primary MET amplification, as a main driver state, has shown a prevalence of 1%–5% in lung adenocarcinomas.

Savolitinib was authorized for use in China in June 2021. It was used to treat NSCLC with METex14 skipping mutations in patients who are refractory to or whose cancer has progressed following platinum-based chemotherapy (7). According to the phase 1b TATTON research, the combination of Osimertinib plus Savolitinib had acceptable safety profile and showed encouraging antitumor efficacy across patients with EGFR mutations, secondary MET amplification, and advanced NSCLC (8). Thus, would the T+S therapy (combined with EGFR-TKI and Savolitinib) be effective in NSCLC patients with primary MET amplification/overexpression and EGFR mutations? Here, we reported 2 cases in which NSCLC patients with primary MET amplification/overexpression and EGFR mutation benefited from T+S therapy.

## Cases

### Case 1

**Figure 1:**
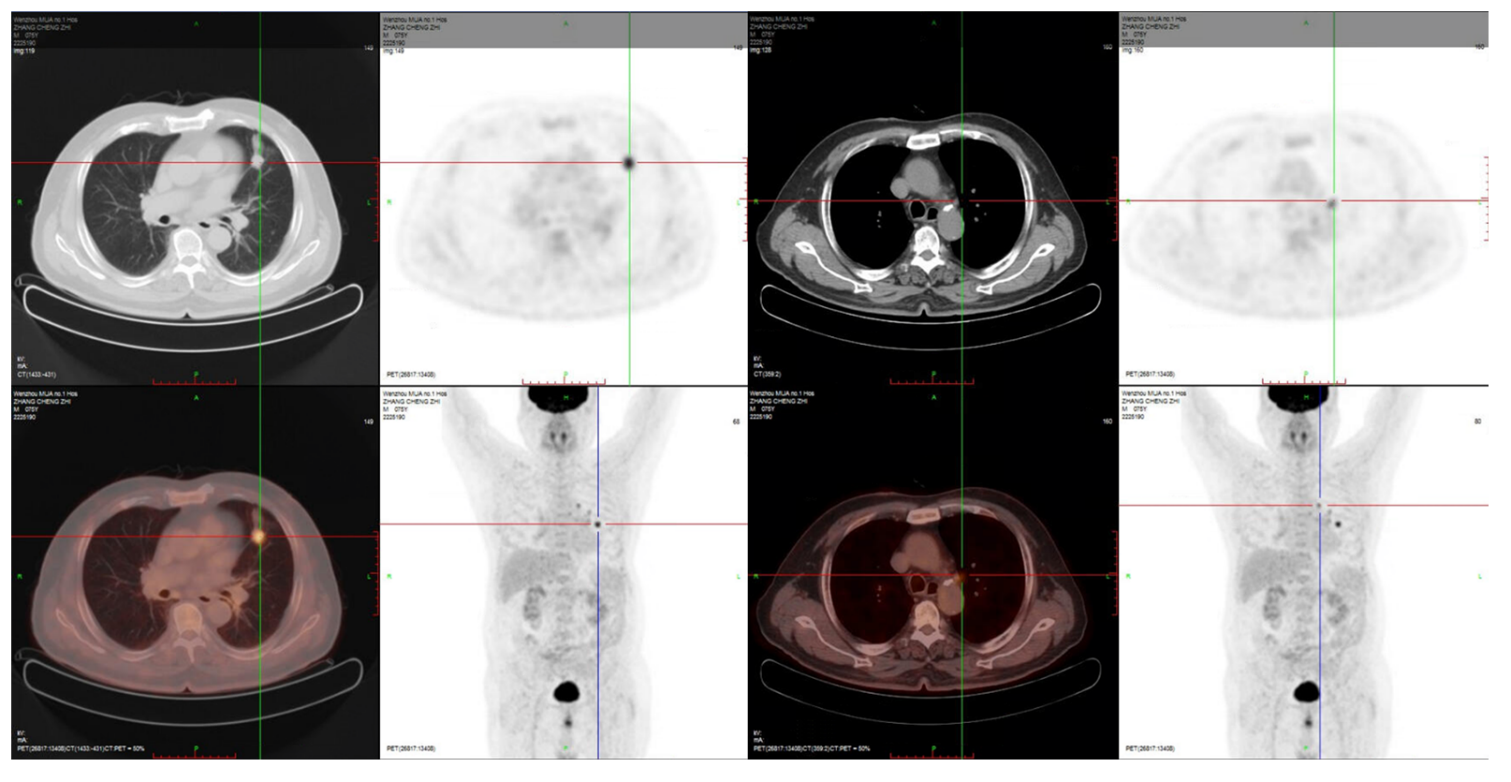
PET-CT on 4 November 2020.

**Figure 2:**
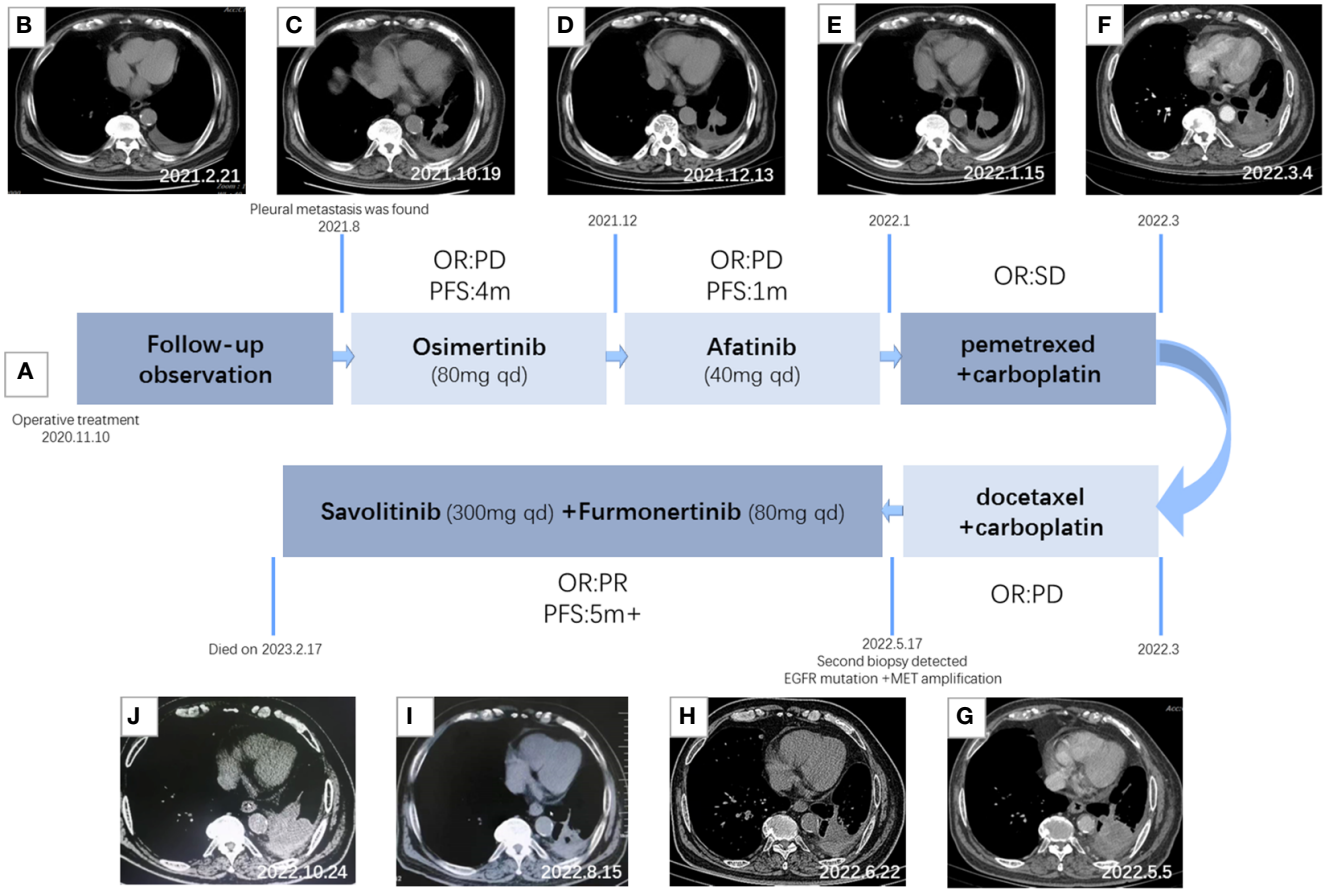
Treatment timeline and clinical response. **(A)** Diagram illustrating the different therapies, including precise timing and therapeutic evaluation. **(B)** CT imaging after surgery. **(C)** CT imaging after 2 months of Osimertinib treatment. **(D)** CT imaging after 4 months of Osimertinib treatment. **(E)** CT imaging after 1 month of Afatinib treatment. **(F)** CT imaging after two courses of combination therapy of pemetrexed and carboplatin. **(G)** CT imaging after two courses of combination therapy of docetaxel and carboplatin. **(H)** CT imaging after 1 month of T+S therapy. **(I)** CT imaging after 3 months of T+S therapy. **(J)** CT imaging after 5 months of T+S therapy.

A 76-year-old man with 50 years of smoking history presented with a hoarseness and pharyngeal foreign body sensation for 1 year. The positron emission tomography (PET) scan on 4 November 2020 showed a hypermetabolic nodule in the left upper lobe with obstructive inflammation, which was considered as pulmonary malignancy first; small lymph nodes in the left upper hilum and mediastinal region 5 suggested the possibility of metastasis (**Figure 1**). Magnetic resonance imaging (MRI) of the brain showed no metastasis. **Figure 2** displays his treatment history and the chest computed tomography (CT) images.

On 10 November 2020, the patient received thoracoscopic radical resection of lung cancer. The pathology after surgery proved that the lesion was a 1 cm * 0.7 cm peripheral invasive adenocarcinoma, without lymph node metastasis and distant metastasis, classified as lung adenocarcinoma (cT1aN0M0, stage IA). Next-generation sequencing (NGS) was performed and the mutations of EGFR p. S768i and EGFR L858R were detected. The patient had a tumor recurrence in August 2021 according to the reviewed chest CT with increased pleural effusion, and there were many nodules on the chest wall that were considered multiple pleural metastases. Then, he had been treated with Osimertinib (80 mg once daily) as first-line targeted therapy since August 2021. After 4 months of targeted therapy, chest CT suggested further deterioration. The treatment regimen had changed several times during December 2021 to March 2022; however, the symptoms had worsened, and his chest pain was so severe that he was confined to bed for extended periods of time, with a PS score of 3.

A CT-guided lung biopsy was performed on 6 May 2022, and NGS analysis of the rebiopsy revealed EGFR L858R and p.S768I mutations. Moreover, fluorescence *in situ* hybridization (FISH) was performed and detected c-MET amplification [cells with MET gene copy number (GCN) >5 accounted for 56%], with a ratio value of 1.18. T+S therapy combined with Savolitinib (300 mg once daily) and Furmonertinib (80 mg once daily) was administrated to the patient on May 19, which had a remarkable effect. After only 3 days of treatment, the chest pain symptom was significantly improved and the PS score was 2. PS score returned to 0 rapidly after 1 month of T+S therapy. A chest CT scan 1 month later demonstrated evident shrinking (PR), and 3 months later, it revealed confirmed PR. CT imaging indicated progress (PD) until 5 months after T+S therapy, and finally, the patient died on 17 February 2023.

### Case 2

**Figure 3:**
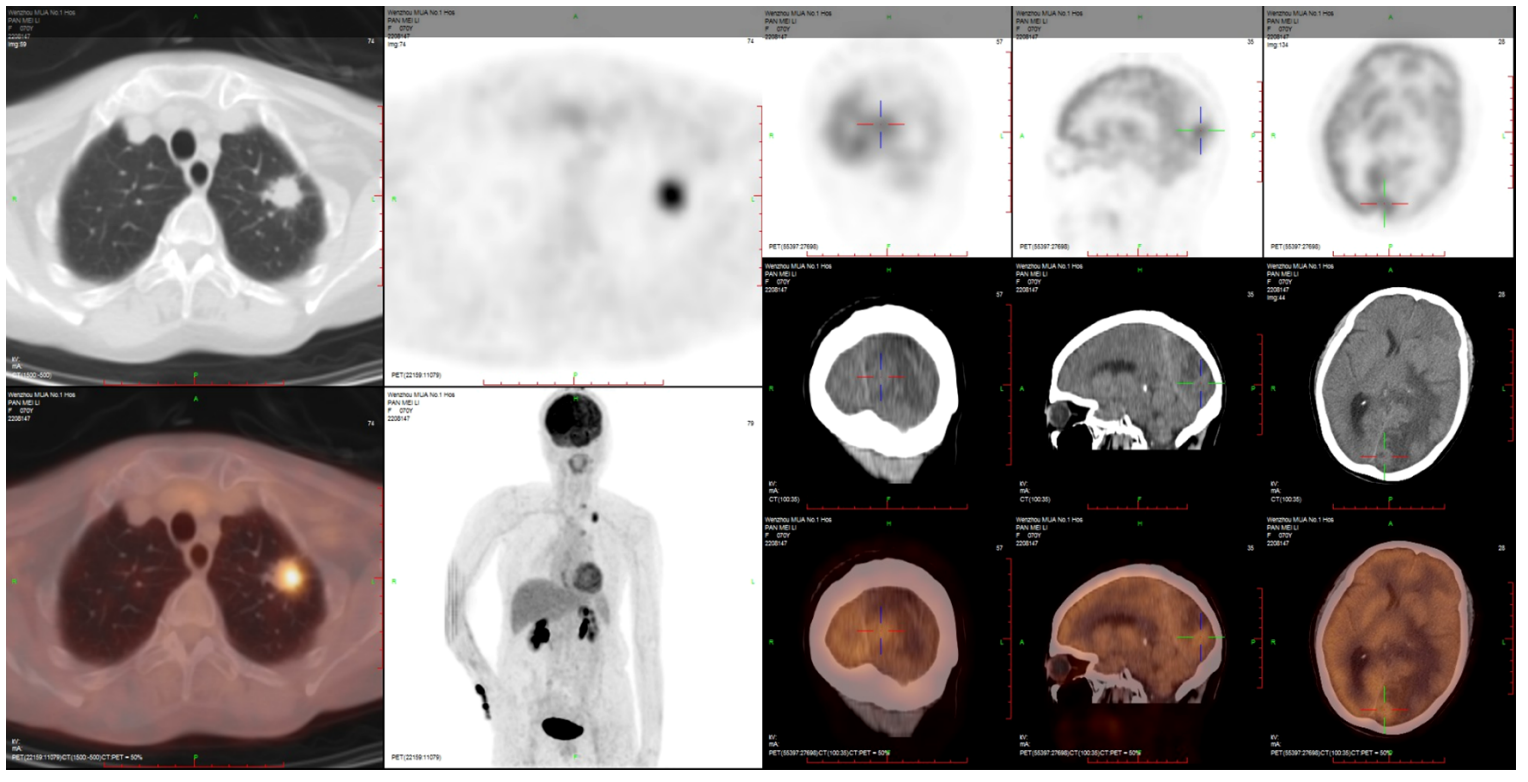
PET-CT on 10 April 2022.

A 70-year-old female never-smoker was admitted to our hospital on 2 April 2022 due to slow reaction and memory decline for 2 months. The brain MRI showed a high possibility of metastasis in the left parietal–occipital lobe, while the chest CT revealed a tumor in the left lung with mediastinal lymph node metastasis. The PET/CT scan is shown in **Figure 3**.

Therefore, the resection of deep supratentorial tumors was performed by a neurosurgeon without any severe complications. The postoperative pathology indicated brain metastatic poorly differentiated adenocarcinoma. Examination of NGS identified EGFR mutations by testing tumor tissue. Based on the findings above, the patient was diagnosed with lung adenocarcinoma (cT1bN2M1b, stage IV). The patient has been treated with Osimertinib (80 mg once daily) as first-line targeted therapy since 6 May 2022. In August 2022, after 3 months of Osimertinib treatment, the chest CT showed that the lung malignant tumor with mediastinal lymph node metastasis was more advanced than before, and the brain MRI revealed a new intracranial lesion, which means Osimertinib had a poor response.

**Figure 4:**
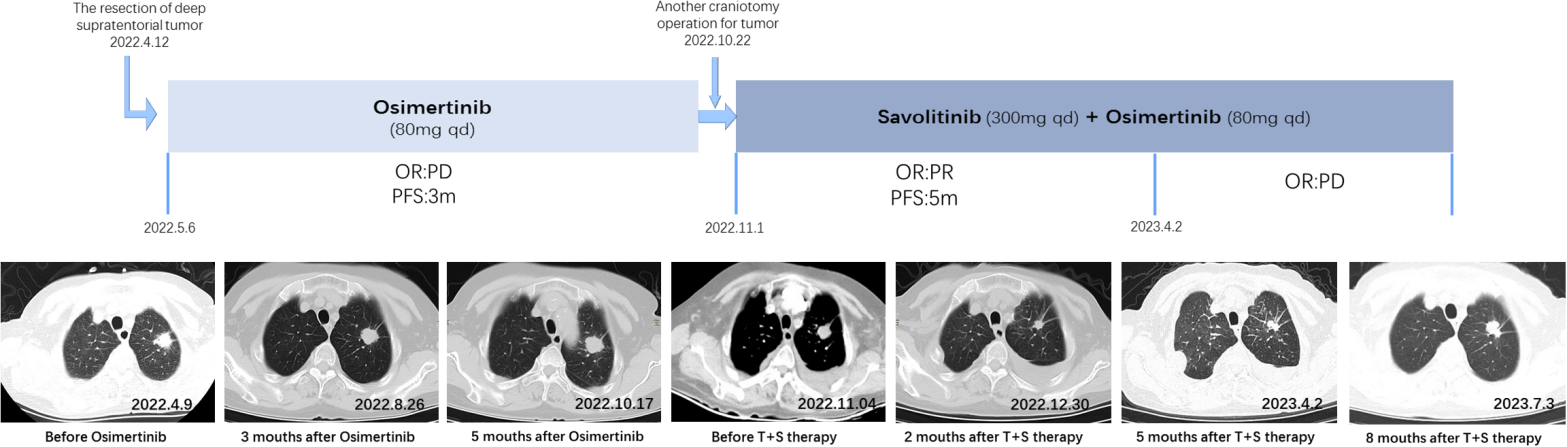
Chest CT imaging of the patient at various time points of the patient’s treatment.

**Figure 5:**
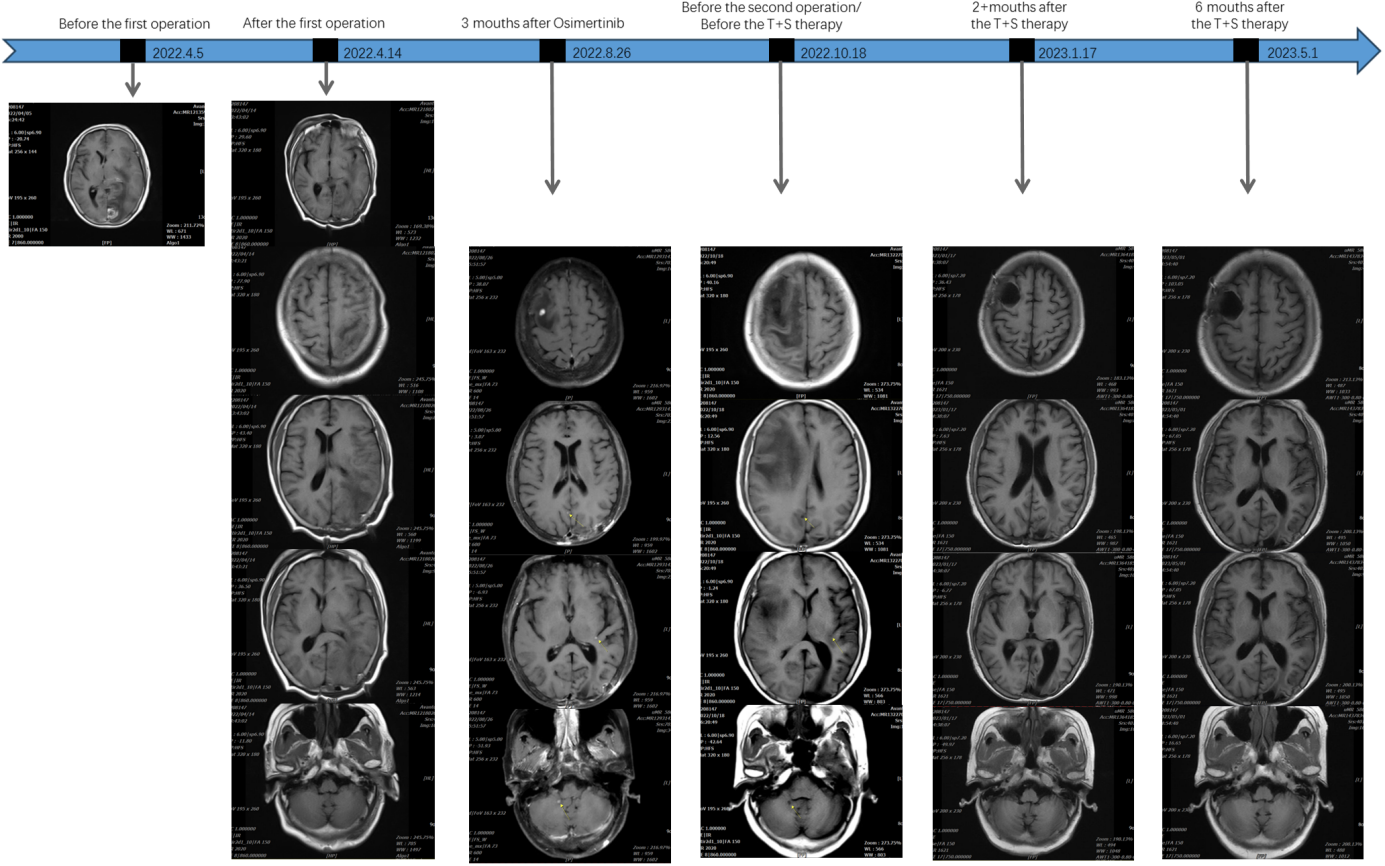
Brain MRI of the patient at various time points of the patient’s treatment.

Because of the further enlargement of the intracranial lesion, another craniotomy operation for the tumor was performed on 22 October 2022. The postoperative pathological finding was also consistent with lung adenocarcinoma metastasis as last time, but this time, we added immunohistochemistry (IHC) for c-MET, and the detection result was c-MET protein overexpression (c-MET 3+). Subsequently, the patient was changed to T+S therapy combined with Savolitinib (300 mg once daily) and Osimertinib (80 mg once daily) in November 2022. The effect of this treatment regimen was remarkable, as the chest CT after 2 weeks showed a reduction in the left lung lesion. The patient remained stable with a progression-free survival (PFS) of 5 months until PD on 2 April 2023. Chest CT imaging and the brain MRI of the patient at various time points before and after treatment are shown in **Figures 4**, **5**.

## Discussion

These two cases described patients with NSCLC, which harbored concomitant EGFR mutation and primary MET amplification/overexpression. Because of the rarity of primary MET amplification/overexpression, the MET target testing is not routinely performed in newly diagnosed NSCLC patients. NGS was used routinely to detect genes in their pathological tissues, and only EGFR mutations were found. First-line therapy with EGFR-TKI did not work, whereas the T+S therapy that combined Savolitinib and EGFR-TKI showed significant efficacy after MET amplification/overexpression was detected by FISH or IHC. There are two categories of resistance to EGFR-TKIs: primary resistance and acquired resistance. Acquired resistance is defined as the progression of the tumor after a period of clinical response, while primary resistance is frequently characterized as a *de novo* inefficacy of EGFR-TKIs (9). The primary resistance occurs through multiple mechanisms such as pre-existing T790M mutations, or the development of off-target genetic alterations including mesenchymal–epithelial transition factor (MET) amplification or HGF mutations (10). The failure of EGFR-TKI as a first-line regimen in these cases suggests abnormal activation of the MET pathway. Under normal physiological settings, the MET proto-oncogene and its ligand HGF (HGF/MET axis) are involved in transduction pathways and influence crucial cellular processes (4). MET activation significantly decreases the efficacy of TKIs due to the crosstalk between the MET and RTK (EGFR) signaling pathways (11, 12). MET amplification can lead to abnormal MET activation by recruiting MET receptor kinase to phosphorylate HER3 and activate the PI3K-Akt survival pathway. Preclinical studies have demonstrated that the coexistence of EGFR mutation and MET amplification/overexpression reduces the sensitivity to EGFR-TKIs, which implicates *de novo* MET amplification/overexpression-mediated primary resistance to EGFR-TKIs (10). It means that only by suppressing EGFR and MET simultaneously may the PI3K-Akt pathway be successfully inhibited (13). In other words, only the combination of MET-TKI and EGFR-TKI would work in advanced NSCLC patients with MET amplification and classical EGFR mutations. In the cases we mentioned, MET target was noticed because of the inefficacy of EGFR-TKIs, and the remarkable efficacy of T+S therapy exactly demonstrated that MET amplification/overexpression was primary.

Savolitinib, approved for use in June 2021 in China, was used to treat NSCLC with METex14 skipping mutations in patients who are refractory to or whose cancer has progressed following platinum-based chemotherapy. This was the first NDA filing in China for a selective MET inhibitor. In the phase 1b TATTON study, Sequist LV et al. (8) concluded that Osimertinib plus Savolitinib might be a potential therapy option for individuals with MET-driven resistance to EGFR TKIs. TATTON is the first initiated clinical study of Savolitinib combined with Osimertinib in advanced NSCLC with MET amplification/overexpression after EGFR-TKI resistance. Patients with locally advanced or metastatic, MET-amplified, EGFR mutation-positive NSCLC, who had progressed on EGFR TKIs, were recruited in this trial, and were divided into two expansion cohorts: parts B and D. The patients in part B were divided into three cohorts: those who had been previously treated with a third-generation EGFR TKI (B1), and those who had not been previously treated with a third-generation EGFR TKI who were either Thr790Met negative (B2) or Thr790Met positive (B3). In part B, patients received oral Savolitinib 600 mg and Osimertinib 80 mg daily. Part D included patients who were Thr790Met negative and had not previously received a third-generation EGFR TKI; these patients received Savolitinib 300 mg plus Osimertinib 80 mg daily. The primary endpoints of the study were safety and tolerability, which were evaluated in all patients given the drug. Secondary endpoints included ORR, DoR, and PFS. With data cutoff on 29 March 2019, 79 (57%) of 138 patients in part B and 16 (38%) of 42 patients in part D experienced adverse events of grade 3 or worse, and serious adverse events (SAEs) were reported in 62 (45%) patients in part B and 11 (26%) patients in part D. The mPFS (median progression-free survival) across all 138 patients in part B was 7.6 months (95% CI 5.5–9.2) with 86 (62%) events. The mPFS was 9.1 months (95% CI 5.4–12.9) with 17 (40%) having a progression event in part D. It indicated that the combination of Savolitinib and Osimertinib has encouraging antitumor activity and acceptable risk–benefit profile. In addition, the better safety of part D compared with part B is the basis for our dose selection of Savolitinib in the two cases above.

Although Savolitinib combined with EGFR-TKI therapy showed dramatic tumor shrinkage in both the primary tumor and brain metastasis to the EGFR mutant NSCLC patients with primary MET amplification/overexpression, the PFS was only 5 months. Such a therapeutic efficacy is not salient in similarly relevant case reports (14), but if MET amplification/overexpression could be accurately detected earlier, perhaps the efficacy of T+S therapy will be better. Consequently, our cases highlight the importance of comprehensive genetic testing in MET for a better choice of targeted therapy. Familiar testing modalities included local tissue FISH, local tissue IHC, or NGS. IHC is for the detection of MET protein overexpression, with the advantages of low price and fast detection speed. FISH is considered the gold standard for the detection of MET copy number alterations (CNAs) including MET amplification (15). NGS is a tissue-saving alternative for detecting diverse genetic aberrations to disclose multiple genes at the same time, and it is particularly accurate in detecting MET exon 14 skipping abnormalities (16).

NGS is widely used in clinical treatment. In order to determine if NGS is a trustworthy technique for the detection of MET GCN, Christoph Schubart et al. (17) conducted a study. NGS and FISH were used to examine FFPE specimens from *n* = 327 consecutive NSCLC cases for genetic abnormalities, including the MET GCN status. Overall, *n* = 205 NSCLC cases could be directly compared using both NGS and FISH regarding the determination of the MET GCN status. Their study indicated that while NGS can detect patients with high-level MET gene amplification, it is inaccurate and fails to detect the various levels of MET gene amplification. NGS cannot replace FISH for the detection of MET GCN.

In the TATTON trial, three test methods—IHC, FISH, and NGS—were adopted to detect MET dysregulation, and the results of the three tests did not overlap completely (18). Preliminary assessment of concordance between MET detection methods from this study showed that subsets of MET-based resistance are detected to different extents through these different testing methods, and these subsets do not completely overlap (19). The SAVANNAH (20), which is an ongoing, randomized, single-arm, global Phase II clinical study, divided patients into groups depending on the level of MET protein in their cancer and/or MET gene according to two tests: IHC (detects if cancer cells have a particular protein or marker on their surface) and FISH (detects a specific DNA sequence from cancer cells), then analyzed how each group’s cancers responded to the treatment combination. SAVANNAH found that patients in the group whose carcinomas had high levels of MET had better outcomes with Savolitinib plus Osimertinib treatment than those with lower levels of MET. Hence, comprehensive and accurate genetic testing is necessary, and the MET gene needs to be detected at first diagnosis for the best choice of targeted therapies. We suggest that it might be effective to combine several test methods for the determination of MET status so as not to overlook patients with MET dysregulation.

Taken together, the idea of combination therapy with EGFR-TKI and MET inhibitor for NSCLC patients with both primary MET amplification/overexpression and EGFR mutation is feasible, and Savolitinib plus EGFR-TKIs should be suggested as a potential effective choice. However, similar cases are rarely reported. To guide precision therapy, further study is required to evaluate whether this kind of treatment modality is the most effective for NSCLC patients harboring concurrent primary MET amplification and EGFR mutation, and it has shown enough promise to be worth looking forward to.

## Conclusion

NSCLC patients with primary MET amplification/overexpression and EGFR mutations are infrequent. According to the two cases above, T+S therapy (dual-target therapy of EGFR-TKI and Savolitinib) has acceptable safety and encouraging antitumor activity for NSCLC patients harboring concurrent primary MET amplification/overexpression and EGFR mutation. T+S therapy has better efficacy and longer survival time compared to the current standard of care for NSCLC patients. On the other hand, a comprehensive and accurate genetic test is necessary, and it will be valuable if the MET gene abnormality is detected at first diagnosis for the optimum option of targeted therapy.

## Data availability statement

The original contributions presented in the study are included in the article/supplementary material. Further inquiries can be directed to the corresponding authors.

## Ethics statement

Ethical review and approval was not required for the study on human participants in accordance with the local legislation and institutional requirements. Written informed consent was obtained from the individuals for the publication of any potentially identifiable images or data included in this article.

## Author contributions

XR: Formal analysis, Investigation, Writing – original draft. KL: Investigation. YZ: Data curation. MS: Conceptualization, Writing – original draft, Writing – review & editing. CZ: Writing – review & editing.
